# Near single-photon imaging in the shortwave infrared using homodyne detection

**DOI:** 10.1073/pnas.2216678120

**Published:** 2023-03-01

**Authors:** O. Wolley, S. Mekhail, P.-A. Moreau, T. Gregory, G. Gibson, G. Leuchs, M. J. Padgett

**Affiliations:** ^a^School of Physics and Astronomy, University of Glasgow, Glasgow G12 8QQ, UK; ^b^Department of Physics, National Cheng Kung University, Tainan 701, Taiwan; ^c^Center for Quantum Frontiers of Research and Technology, National Cheng Kung University, Tainan 70101, Taiwan; ^d^Max Planck Institute for the Science of Light, Erlangen D-91058, Germany; ^e^Department of Physics, Friedrich-Alexander-Universität Erlangen-Nürnberg, Erlangen 91054, Germany

**Keywords:** optics, imaging, microscopy

## Abstract

Low-light imaging is challenging in regimes where low-noise detectors are not yet available. One such regime is the shortwave infrared where even the best multipixel detector arrays typically have a noise floor in excess of **100** photons per pixel per frame. We present a homodyne imaging system capable of recovering both intensity and phase images of an object from a single frame despite an illumination intensity of **≈**​**1** photon per pixel. We interfere this weak signal which is below the noise floor of the detector with a reference beam that is **∼**​**300,** **000** times brighter, record the resulting interference pattern in the spatial domain on a detector array, and use Fourier techniques to extract the intensity and phase images. We believe our approach could vastly extend the range of applications for low-light imaging by accessing domains where low-noise cameras are not currently available and for which low-intensity illumination is required.

## Significance

While low-light imaging has become crucial in many areas, applications are still limited to the visible region of the spectrum where detector noise performance is favorable. We present a full-field, homodyne imaging system capable of imaging at the single-photon level in the shortwave infrared, despite the camera having a noise floor ≈200 times higher. This interference approach to low-light imaging was first suggested by Gabor 70 y ago. Our approach requires only a single camera frame, unlike other temporally based interference methods which require temporal scanning. While extending the capacity of holographic microscopes, our demonstration will also extend the range of applications of low-light imaging, benefiting biological, medical, and covert imaging.

Techniques to perform imaging under a low-photon flux are important in various contexts, such as medical and biological imaging, where optical exposure has to be minimized and covert imaging where a low-photon illumination flux is required. As the illumination level is reduced, the dark noise and electronic readout noise of the detector becomes an important consideration and degrades the resulting image quality. Once the detected signal is of the order of the detector noise, it is no longer possible to distinguish the object being imaged from the background. While for visible wavelengths the detector noise is typically equivalent to a single photon per pixel per frame, in other regions of the spectrum such as the shortwave infrared (SWIR), detector noise is much more significant. In the SWIR, nonsilicon-based detectors such as those based on indium gallium arsenide (InGaAs) are used which have noise floors at least an order of magnitude higher than an equivalent silicon-based detector in the visible region of the spectrum. Increasing the illumination level overcomes the high detector noise; however, this is not always possible in low-light imaging contexts in which the photon flux must be minimized. For example, in the context of biological imaging applications, where illuminating a live sample with a high probe beam power may alter cell processes and a high probe beam power can lead to cell death (1, 2). In absorption measurements, increasing the probe power can also lead to saturation of the sample (3). Further to these applications, low-light imaging is desirable for LiDAR and covert imaging where either low-power eye-safe sources are required or in cases where imaging is performed through scattering media (4, 5).

Various interference-based methods have been demonstrated as ways of performing sensitive measurements. Nonlinear techniques such as frequency upconversion and nonlinear interferometry are capable of recording sensitive measurements at infrared wavelengths on visible detectors (6, 7). Homodyne and heterodyne detection have also been widely used to measure with a sensitivity below the noise constraints of a system across various applications (8–15). Using homodyne measurements, where the interference of two waves originating from the same source is measured rather than a direct signal, one can measure with increased sensitivity and have access to both amplitude and phase information of an unknown wave front. For example, homodyne measurements reveal phase singularities (16), potentially leading to superresolution imaging (17). In our work, we concentrate on the optical amplifying aspects of homodyne measurements to enable imaging below the noise floor of an imaging detector. We also note that by using homodyne or heterodyne detection, it is in principle even possible to obtain information about the spatial pattern imprinted on a single photon in a single measurement with a signal-to-noise ratio of one (18, 19).

Our technique has parallels with both digital holographic microscopy (DHM) and full-field optical coherence tomography (OCT). DHMs interfere the signal beam with a reference beam to recover both the intensity and phase of a transmissive sample (20–23). However, the signal and reference beams are routinely set to have a comparable intensity, thereby maximizing the fringe contrast. OCT relies on the interference between a reference and a much weaker backscattered signal beam. OCT typically uses a short coherence length source to measure both depth and intensity, albeit usually in a raster or line-scanned configuration. The interference is usually measured in the temporal domain through either deliberate phase stepping or heterodyne modalities (24–26).

Our scheme is different from both conventional DHM and OCT schemes in that we deliberately unbalance the signal and reference beams and utilize spatial interference by performing off-axis holography to collect full-field images within a single detector frame. The interference fringes have a visibility scaling with the square root of the intensity ratio between the signal and reference beams. This scaling allows us to measure the signal beam despite its intensity being significantly below the noise floor of the camera. In applying these techniques, we have realized one of the initial suggestions of Gabor for imaging without (or more accurately with minimal) illumination (27). One previous implementation has also achieved full-frame real-time imaging by an unbalanced interferometer via DHM (28). In that work, a standard intensity reconstruction was compared to regularized intensity image reconstruction from a detected interference pattern to investigate compressed sensing techniques with regard to holography as have others in the field (28–31). In contrast to our work this experiment was conducted in the visible regime where single-photon–sensitive cameras are already available. Here, we demonstrate intensity and phase imaging under the conditions of a signal beam intensity which is 1/200th of the noise floor of a SWIR camera and compare to the conventional images under a range of illumination levels, achieving significant improvements in sensitivity and resulting image contrast.

In relation to other work, a homodyne detection scheme has been implemented in the context of quantum imaging in which squeezed states of light were used to probe an object and image below the noise floor of the detector by interfering a signal with a local oscillator (32). The authors have also generalized their system to work with thermal states, obtaining shadow images of a semitransparent object by measuring the mean temporal variance between the two output ports of the interferometer (33). The method of using an unbalanced interferometer has also been used to measure orbital angular momentum modes of light in a low-illumination regime (34). Other low-light DHM imaging systems include those that implement computational methods (35, 36). While we are unable to image at such low photon counts as a quantum scheme, our classical imaging technique is capable of real-time imaging from a single frame with the signal beam below the noise floor of the detector without the requirement of a quantum source. The ability to obtain these images with the signal beam below the noise floor of the detector in a single frame is useful for the imaging of moving objects or when using short exposures. Furthermore, we obtain both intensity and phase images of an object allowing for improved sample inspection.

The method of performing a homodyne measurement to extract both the intensity and phase information of a sample has been applied in biological imaging for cell imaging and disease identification (37–39). Label-free biological imaging methods have the advantage of not being chemotoxic or causing modifications to cell chemistry which can kill the sample or interfere with the observation of cell processes (40). Our method could be further adapted to nondestructive testing and inspection applications that require imaging at low-illumination levels or at wavelengths for which detectors have a high noise floor due to readout and environmental noise such as is the case for detectors operating in the far-infrared or terahertz. Furthermore, in the context of medical imaging, patient dose is an important consideration so applying this technique to medical imaging schemes to amplify a weak signal that has been used to probe the patient with a strong local oscillator in order to limit patient dose. Other diffractive imaging techniques, such as Fourier ptychography, could also serve in some of these contexts for the purpose of obtaining phase images (41, 42). Further interference-based imaging schemes, such as those that utilize multiple wavelengths, could also benefit from this technique (43, 44).

In this work, we present a homodyne detection system that is capable of imaging underdetected signal beam illumination levels that are below sensor readout noise in the shortwave infrared wavelength regime. We are able to recover intensity and phase images where features of the object are distinguishable down to a signal intensity of ∼1.1 photons per pixel incident upon the camera sensor, around 200 times below the noise floor of the camera. We are able to recover a measurable image contrast for the homodyne interference image as compared to a wide-field image where noise dominates over the image contrast. This enhancement in image contrast for the homodyne image relative to the direct image has a corresponding reduction in image resolution of 29.2%. We believe our demonstration shows the possibility of low-light imaging in domains where detector noise is a significant issue, extending the range of low-light imaging applications.

## Methods

### Imaging System.

**Fig. 1.**
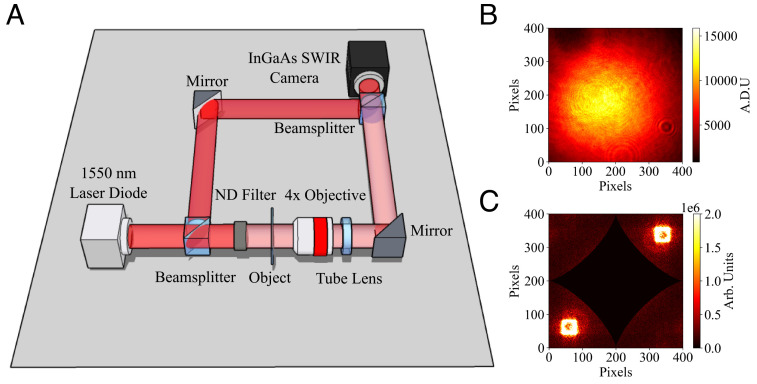
(*A*) Diagram of the experimental method setup to obtain interference images. We use a microscope built in a transmission configuration using a 4x microscope objective and 150 mm tube lens to image the object onto the camera. To perform an optical homodyne measurement, we split our illumination source using a beam splitter. The signal beam passing through the object is attenuated to a low intensity by the use of a neutral density (ND) filter before being interfered at the second beam splitter with a reference beam of much greater intensity. (*B*) Example hologram recorded on the detector showing low fringe contrast due to the unbalancing effect. (*C*) Example of a FFT of an obtained hologram, with the central DC components masked out by a curved diamond shape.

An inverted wide-field microscope was built with a secondary reference arm as presented in the experimental setup in Fig. 1. The laser used is a 1,550- nm laser diode with a coherence length of 24.0 mm. To perform an optical homodyne measurement, the laser beam is split into the signal arm, and the reference arm at the first beam splitter. The object is placed into the signal arm, and the relative intensity of the two beams is controlled using reflective neutral density (ND) filters, with a more optically dense filter in the signal beam such that the reference beam has a higher intensity. The signal and reference beams are then recombined at a second beam splitter, and the object imaged onto the camera detector array.

The camera used is a InGaAs SWIR camera with a typical readout noise of 180*e*^−^ per pixel and quantum efficiency (QE) of 85% at 1550 nm. Taking this QE into account, the effective noise floor of the camera is ∼​207 photons per pixel. We use the low-gain mode of the sensor, which while noisier gives a higher dynamic range to allow for a greater ratio between signal and reference beams.

### Intensity and Phase Reconstruction.

We obtain an intensity image of the interfered signal and reference beams on our camera, which contains information about the signal and reference fields *E*_sig_ and *E*_ref_ according to

[1] $$\begin{array}{c} \begin{array}{cc} I_{\;\text{tot}} & \propto |E_{\;\text{sig}}\left(r\right)+E_{\;\text{ref}}\left(r\right)\;\text{exp}\left(ik_{\mathrm{tilt}}r\right){|}^{2}\\ & =I_{\;\text{sig}}+I_{\;\text{ref}}+2\;\text{Re}\left[E_{\;\text{sig}}\left(r\right)E_{\;\text{ref}}\left(r\right)\;\text{exp}\left(ik_{\mathrm{tilt}}r\right)\right], \end{array} \end{array}$$

where **k**_**tilt**_ is the relative wavevector between the propagation of *E*_sig_ and *E*_ref_. We take an average of the reference beam over a minimum of a hundred frames and subtract this from the hologram. *E*_ref_ is assumed to have a flat phase front which is fixed by proper alignment of the reference beam, and the field is therefore estimated as $\sqrt{I_{\;\text{ref}}}$. In a method similar to that of Fatimi and Beadie (45), we take a FFT of *I*_tot_ − *I*_ref_. Note that in order to extract the field of the signal beam (*E*_sig_) from the detected intensity, the subtraction of *I*_ref_ is necessary as shown by Eq. **1**. This is especially important in the unbalanced regime where *I*_sig_ ≪ *I*_ref_ as the DC Fourier component of the reference beam *I*_ref_ cannot be simply masked out as it does not comprise a single point but has a tail which extends out to the portions of spatial-frequency space that contain the interference signal. Since *I*_sig_ ≪ *I*_ref_ and is also below the noise floor of the camera, it is simply neglected. Any remaining low-spatial-frequency components of the FFT are masked as per Fig. 1*C*. One of the four quadrants is selected to highlight the location of the interference pattern and the other three are masked. For example, as can been seen in Fig. 1*C*, two bright areas are present in diagonally opposing quadrants. The selection of the wrong one simply gives the conjugate phase of the object being imaged. Determining which quadrant corresponds to the relative tilt between the reference and signal arms can be performed with a reference sample. Once the unused quadrants are masked, the relative wavevector **k**_**tilt**_ is determined by an algorithm based on a center of mass calculation. This is used to approximate the relative reference field as $\sqrt{I_{\;\text{ref}}}e^{ik_{\mathrm{tilt}}r}$. The masked FFT then undergoes an inverse FFT, and the resulting complex field is pointwise coherently divided by the relative reference field. The result is the complex field image of the sample which is then split into modulus and argument to give the transmissive amplitude and phase profile of the sample, respectively.

We confirmed the linearity of the gray scale between a conventional image and a homodyne intensity image by imaging a ND filter where half the frame was covered by the filter and the other half-clear glass and ensuring the gray scale values between the two regions dropped by an equivalent amount for both the conventional wide-field image and homodyne image.

Due to physical constraints of the microscope, the signal arm introduces a spherical phase front not present in the reference arm. As such, this is measured and subtracted from the calculated phase profile of the sample. Due to the instability of the system on a frame-by-frame basis, we apply temporal averaging on our reference phase image such that errors are not incurred during the subtraction. The reference phase does not need to be remeasured for every new object, as it is a correction for the optical path difference of the optics in the system. When it has been recorded, the samples can then be introduced or interchanged, and no further calibration is needed.

## Results

**Fig. 2.**
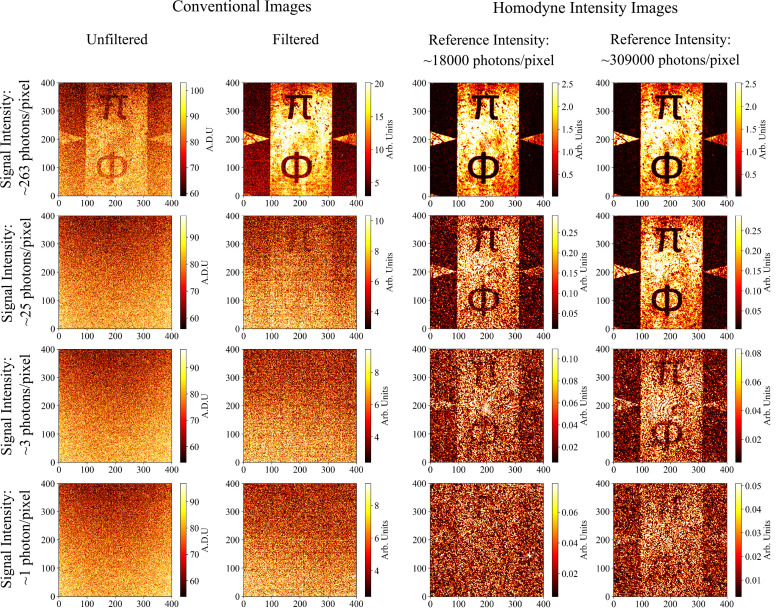
A table comparing conventional camera images and reconstructed homodyne intensity and images at differing illumination levels. Shown are conventional camera images at four different signal intensities incident on the camera alongside the corresponding reconstructed intensity and phase images from the homodyne system of a silicon chip with gold-deposited features. The reconstructed images are shown using a low-intensity reference corresponding to a detected intensity of 18,000 photons per pixel and also a high-intensity reference beam corresponding to an intensity of 30,9000 photons per pixel. Due to spatial filtering inherent in the homodyne image reconstruction process, we also show conventional images with a background noise subtraction and equal degree of spatial filtering applied alongside the raw images for fair comparison. All conventional and homodyne intensity images were normalized by setting the minimum and maximum of the scale to the value of the 10th and 90th percentiles of pixel values, respectively.

A series of images of a silicon chip with gold-deposited features acquired by the system under a range of different illumination scenarios are shown in Fig. 2. The silicon regions are transparent to light in the SWIR, while the gold regions reflect the light. Conventional images of the signal beam are shown at four different illumination levels along with homodyne intensity images reconstructed from holograms recorded at low and high reference beam intensities corresponding to detected intensities of 18,000 and 30,9000 photons per pixel, respectively. As the reconstruction of intensity images involves spatial filtering (masking in the Fourier plane) of the DC components, we also show filtered images where we perform a background noise subtraction and apply an equivalent amount of spatial filtering for fair comparison. All images shown are taken from a single camera frame. We calculate the intensity of the reference beam detected on the camera by converting the pixel values recorded into a number of photons using the stated full-well capacity of the pixels and quantum efficiency of the sensor at 1,550 nm. We are unable to use this method to calculate the signal intensity at the camera, as at lower illumination levels, the signal information is contained within one bit, so we cannot convert accurately to a photon number. Instead, we calculate the ratio between the two arms and use the calculated reference intensity to obtain a value for the intensity of the signal arm. In order to do this, we use the manufacturer stated transmission at 1,550 nm of the ND filters used, and measure the drop in power across the objective and tube lens using a power meter. The intensity of the signal arm is the same for both the conventional image and its corresponding reconstructed image.

It can be seen from Fig. 2 that the homodyne system is capable of recovering contrast of the object in the signal arm by either reducing noise on the image when the object is partially visible or even recovering features of the object when the image contrast is dominated by camera noise. In the bottom row of Fig. 2, we present an image obtained for a single frame with an estimated signal intensity of 1.1 photons per pixel per frame. This means that for an effective noise floor of ∼​207 photons per pixel, we are able to obtain intensity from a single camera frame with no requirement to perform pixel binning in postprocessing under an illumination level ∼​200 times below the detector noise.

**Fig. 3.**
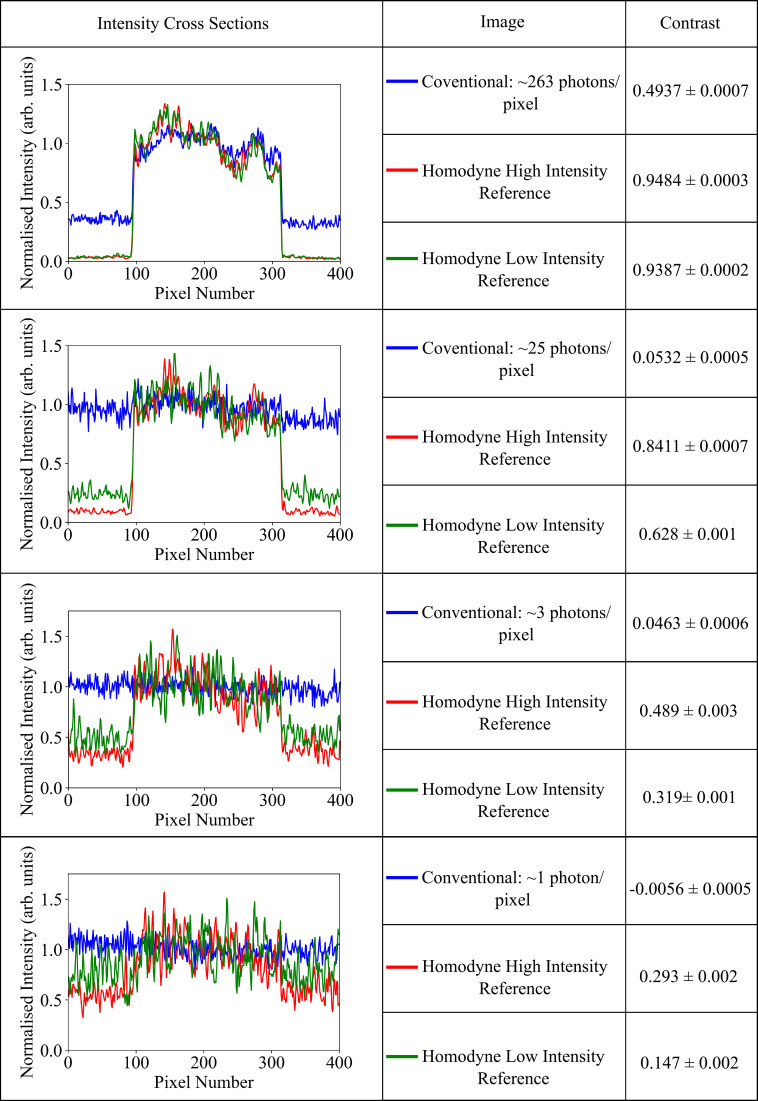
Image intensity cross-sections for the images displayed in Fig. 2 alongside image contrast statistics. Image intensity cross-sections were produced by averaging image intensity over columns between two sets of rows for each image (conventional camera image, homodyne intensity at a high-intensity reference, and homodyne intensity at a lower intensity reference). Columns 0 to 400 were averaged over between rows 10 to 45 and 230 to 265 to generate the intensity cross-section line plots. These plots were displayed on the same scale by normalizing each to the average value of the bright regions of the cross-section, columns 100 to 300. The contrast was then determined using the average value of these bright regions and the average value of the dark regions and averaged over 100 frames with the SE on the mean calculated. The dark regions were determined by columns 0 to 75 and 325 to 400.

The performance of the system is linked to the intensity of the reference beam. If the intensity of the reference beam is lowered, the signal is not amplified to as greater an extent, and the recovered homodyne images are noisier and have reduced contrast. This can be seen in Fig. 2 and is further illustrated in Fig. 3 showing intensity cross-section plots of the images in Fig. 2 alongside calculated values of the contrast for each image. Intensity cross-sections for the homodyne and signal images are taken by averaging over columns between two sets of rows with clear bright and dark regions, rows 10 to 45 and 230 to 265. They are then displayed on the same plot by normalizing to the average value of the bright regions in each cross-section. The contrast was calculated as (*I*_bright_ − *I*_dark_)/(*I*_bright_ + *I*_dark_), where *I*_bright_ and *I*_dark_ are the average intensity values for the bright and dark regions of the image, respectively. This was averaged over 100 frames with the SE on the mean also calculated. It can be seen particularly at the lower signal intensities that image contrast is reduced for a lower intensity reference beam, due to the interference term in the intensity profile recorded on the camera not being amplified to as greater extent. At these lower reference beam intensities the detector noise becomes significant and the images obtained have reduced contrast, demonstrating the importance of a high powered reference to preserve image contrast.

It can also be seen from Fig. 3 that as the signal intensity decreases, the contrast of the recovered homodyne images decreases. While the homodyne method is able to decrease the contributions of detector noise, it cannot eliminate shot noise arising from the fluctuations of light itself. These fluctuations will make a contribution in the subtraction of the reference beam as well as on the signal itself. As the intensity of the signal decreases, the signal-to-noise ratio decreases, and there is reduced contrast in the recovered homodyne images. At an illumination level of ∼​1.1 photons per pixel, while the contrast is reduced to 0.293 ± 0.002, the contrast in the corresponding conventional image is completely dominated by noise, measured as being −0.0056 ± 0.0005. This means features of the object can be distinguished using the homodyne imaging method which would otherwise not be observable with simple conventional imaging.

**Fig. 4.**
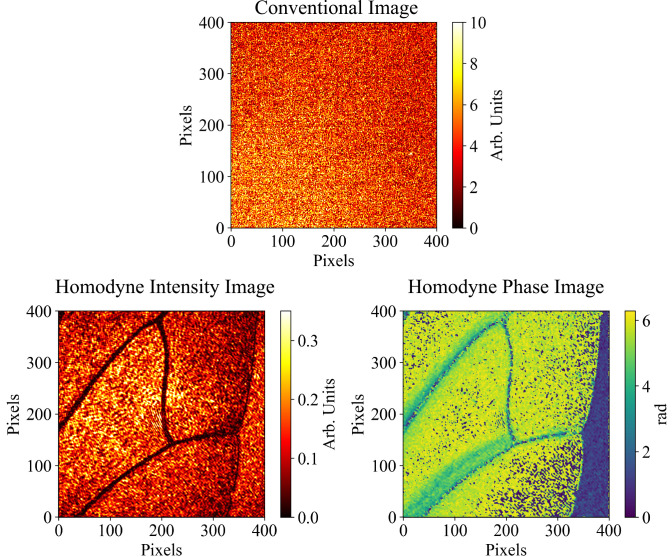
Single-frame conventional camera image alongside corresponding homodyne intensity and phase images of an insect wing. Homodyne intensity and phase images show features of a transmissive object can be recovered by the system when they are not visible in a conventional camera image. Furthermore, access to the phase image gives information on the optical path difference caused by the object. Note the blue portions toward the lower right of the wing showing the phase discontinuity where the optical path difference exceeds one wavelength. The intensity of the signal beam illumination incident on the camera was calculated to be ∼​25 photons per pixel.

We also present the imaging of a transmissive object, and the recovery of phase and depth information from the sample when the illumination level is below the camera noise. Fig. 4 shows an image of an insect wing at an illumination level of ∼​25 photons per pixel from a single camera frame. Shown is the direct signal image along with intensity, phase, and depth information recovered from the hologram. From the phase image, it is possible to convert the scale into depth information in μm. Access to depth information allows for greater inspection of a transmissive object as it can be seen from the scale in the phase image that the wing gets thinner toward the vein areas. The ability to record phase and depth information would be attractive in the imaging of biological and material samples as it can reveal features that are otherwise not visible from an intensity profile alone at a sensitivity level not accessible without homodyne amplification.

With our system, we observe a loss in resolution of the reconstructed images when compared to the conventional camera image. While the Nyquist theorem states the fundamental limit of holography systems is two pixels (to observe a bright or dark fringe), in principle, the optical design of microscopes to be oversampled at the detector means this limit is not reached. Instead, the drop of resolution observed in our system is due to the masking of spatial frequencies in the Fourier plane. We argue however that this could also be overcome by optical design. If the wavevector **k**_**tilt**_ is large enough, then we could sufficiently separate the information contained by the interference fringes from the central DC components in frequency space and, thus, masking DC components would not lead to any spatial frequencies corresponding to image information being lost. In practice, this would be achieved by **k**_**tilt**_ being larger than the numerical aperture (NA) of the system at the detector, given by the NA of the microscope objective divided by the magnification. In our system, using a low magnification objective, we cannot achieve a value of **k**_**tilt**_ larger than the NA at the detector due to the pixel pitch of the camera. However, with a higher-magnification objective or smaller pixel pitch, it should be possible to obtain fringes with **k**_**tilt**_ larger than the NA at the detector and not observe a drop in resolution in the reconstructed homodyne images when compared with the conventional image. We also note a modification of this experimental configuration to enable access to a range of different **k**_**tilt**_ angles by angular shaping of the reference beam could enable superresolved imaging to be performed similar to Fourier ptychography setups (46).

**Fig. 5.**
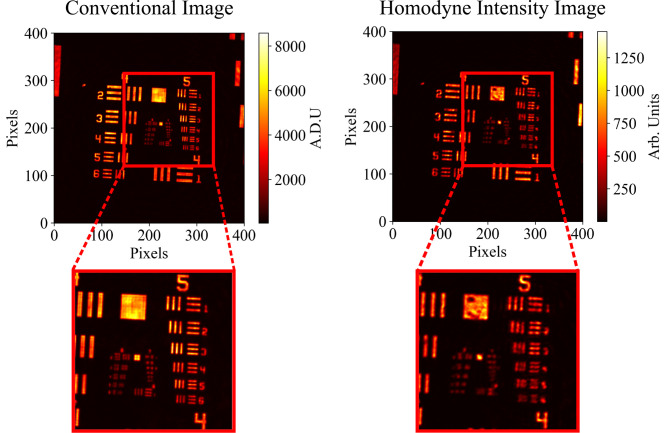
Images of a USAF resolution target from the conventional camera image and reconstructed homodyne intensity image. It can be seen that in the conventional camera image, the smallest resolvable element is 1 in group 6 (largest element in the lower left of inner square), while for the homodyne image, it is element 4 of group 5 (fourth element down on the right-hand side). This corresponds to a loss in resolution of 29.2% when images are reconstructed using the homodyne method when compared with what could be achieved with a conventional camera image.

In order to quantify the resolution loss of our experimental setup, a raw image of a USAF resolution target is compared with its corresponding homodyne intensity image in Fig. 5 to calculate the loss of resolution in the obtained homodyne images. It can be seen from Fig. 5 that in a conventional image taken with the system, element 1 of group 6 is resolvable, giving a resolution of 64.0 lp mm^-1^ (line pairs per millimeter). We see in the corresponding homodyne image that element 4 of group 5 is the smallest resolvable element, giving a resolution of 45.3 lp mm^-1^. This means with our implementation of the homodyne method, there is a 29.2% drop in resolution in the corresponding intensity and phase images. With some trade-off between noise sensitivity and resolution, we chose a mask size which preserves noise sensitivity without losing too much resolution and used a shape of form (*x*^*p*^ + *y*^*p*^)^(1/*p*)^ = *r*, which for a value of *P* = 0.75 gives a curved diamond shape. The radius of the mask used was 200 pixels. While there is a modest loss in resolution, we anticipate the homodyne method being most useful in situations where features of the object would otherwise be obscured by noise and not resolvable due to required low-illumination levels.

## Discussion

We have presented a homodyne detection method capable of reconstructing intensity and phase images below the camera noise floor at a range of different illumination levels, with image contrast increased down to a detected signal intensity of 1.1 photons per pixel. This shows the ability of the system to image under the conditions of low sample illumination below the noise floor of the detector. With a camera with an effective noise floor of ∼​207 photons per pixel, we are able to image under the conditions of a signal intensity ∼​200 times lower than the noise floor of the camera. With our imaging system, a degradation in resolution of 29.2% is observed; however, we note that this loss in resolution is not inherent to the method but due to the imaging system used. The system can also image transmissive objects and recover intensity, phase, and depth information about the object, being able to determine the thickness of a sample. The enhancement in sensitivity is made possible because homodyne detection amplifies the optical signal before the photoelectric conversion process at the detector.

We have identified the intensity of the reference beam to be a key parameter to the performance of the system, with a lower reference intensity leading to reduced contrast in recovered intensity images. As such, the dynamic range of the camera is an important consideration when designing the system. We report recovering homodyne intensity and phase images at a maximum ratio between signal and reference beams of ∼​300,000:1 using a camera with a dynamic range of 72 dB.

With the ability to perform a wide-field imaging protocol in real time with signal intensities below the noise floor of the detector, we believe our system could find applications in low-light imaging scenarios where low-noise detectors are not currently available. With the system being applicable to a broad wavelength range, it could vastly expand applications in low-light imaging contexts. Here, it could be used to either decrease acquisition times to obtain images of equivalent contrast at faster speeds or to allow imaging at illumination levels where it would otherwise not be possible to determine the features of an object.

## Materials and Methods

The infrared light source at 1,550 nm is a Thorlabs LP1550-PAD2 with an actual center wavelength of 1548.7 nm and a typical linewidth of 0.1 nm, giving a coherence length of 24.0 mm. The SWIR infrared camera is a Raptor Owl 640M SWIR camera with an InGaAs sensor of 15μm × 15μm pixel size. The noise floor of the camera was stated to be 180*e*^−^ per pixel. The well-depth of the camera in the low-gain mode is stated to be 650, 000*e*^−^. With the sensor having a quantum efficiency (QE) of 85% at 1,550 nm, 1*e*^−^ = 1.18 photons, we calculated the effective noise floor to be ∼​207 photons per pixel. The power meter head used to determine the absolute and relative powers of the signal and reference beams in this experiment is a Thorlabs S122C. The estimated proportion of light at 1,550 nm that passes through the microscope objective and tube was determined to be ∼56% using the 1,550- nm laser and measuring the power before and after the optics. The sample illumination was determined by measuring the power after the first beam splitter and the manufacturer stated transmissions of the ND filters and using a measured beam diameter of 3 mm. The ND filters used were Thorlabs NEIRxxA ND filters, and the manufacturer stated transmissions were used in calculating the power of the signal beam. ND filters and transmissions for each dataset presented in Fig. 2 are as follows: NENIR05A-C - 34.24%, NENIR20A-C - 1.2818%, NENIR30A-C - 0.1472%, and NENIR40A-C - 0.0144%.

## Data Availability

Image data to generate figures data have been deposited in University of Glasgow Library Data Repository (http://dx.doi.org/10.5525/gla.researchdata.1388).
